# Immunotherapy or antiangiogenic therapy plus chemotherapy as first‐line treatment of patients with PD‐L1(‐) advanced non‐squamous non‐small cell lung cancer in a Chinese cohort

**DOI:** 10.1002/cam4.6101

**Published:** 2023-05-22

**Authors:** Ruolan Xia, Yanying Li, Ling Yang, Meijuan Huang

**Affiliations:** ^1^ Department of Biotherapy, State Key Laboratory of Biotherapy West China Hospital, Sichuan University Chengdu Chengdu China; ^2^ West China School of Medicine Sichuan University Chengdu China; ^3^ Division of Thoracic Tumor Multimodality Treatment and Department of Medical Oncology, Cancer Center West China Hospital, Sichuan University Chengdu China

**Keywords:** bevacizumab, chemotherapy, immunotherapy, nonsquamous non‐small cell lung cancer, treatment

## Abstract

**Purpose:**

For patients with advanced nonsquamous non‐small cell lung cancer (NSCLC), immunotherapy or antiangiogenic therapy combined with pemetrexed and cisplatin/carboplatin have both shown significant efficacy at programmed cell death ligand 1 (PD‐L1) levels of <1%. Our study aimed to compare two first‐line regimens for patients with advanced nonsquamous NSCLC who were negative for PD‐L1.

**Methods:**

A retrospective cohort study was conducted comparing the outcomes of patients with advanced PD‐L1(‐) nonsquamous NSCLC who were treated with antiangiogenic therapy plus chemotherapy (A Group) to those who were treated with anti‐PD‐L1 monoclonal antibodies plus chemotherapy (mAbs) (B Group). Both regimens were evaluated for progression‐free survival (PFS), overall survival (OS), objective response rate (ORR), disease control rate (DCR), and side effects.

**Results:**

114 patients were enrolled in the study, 82 in Group A and 32 in Group B. Those in Group A had a longer median PFS (9.8 vs. 6.7 months, *p* = 0.025). The OS was also achieved (*p* = 0.058). No statistically significant difference was seen in ORR (52.4% vs. 50.0%, *p* = 0.815) or DCR (93.9% vs. 87.5%, *p* = 0.225) between the two groups. Patients in the A group who did not smoke and did not have specific metastases could benefit from survival. Adverse events (AEs) in both groups were tolerated.

**Conclusion:**

Bevacizumab plus chemotherapy outperformed immunotherapy plus chemotherapy in terms of PFS.

## 1 INTRODUCTION

A large number of lung cancer cases and deaths occur every year across the globe, with an estimated 2.20 million new cases and 1.79 million deaths annually.^1^ The main risk factor is smoking, along with other exogenous and endogenous factors that contribute to individual risk, such as diet, genetic predisposition, preexisting lung disease, and environmental factors.^2^, ^3^, ^4^ The main pathological type of lung malignancies is non‐small cell lung cancer (NSCLC), accounting for 85%–90% of cases, and NSCLC can be divided into squamous and nonsquamous subtypes. A majority of patients miss the opportunity to undergo surgery due to advanced stages of the disease at the time of diagnosis. For patients with metastatic NSCLC. The last decade has witnessed significant progress that has led to substantially improved survival. The first‐line therapeutic regimens are now recommended for advanced patients without oncogene‐driven NSCLC, including pembrolizumab, atezolizumab, and cemiplimab monotherapy (PD‐L1 ≥ 50%), chemotherapy alone, or combination therapy based on platinum‐based chemotherapy.^5^

In particular, a substantial survival benefit has been achieved through the development of related antibodies against programmed cell death protein 1 (PD‐1) and its ligand (PD‐L1) for a proportion of these patients.^6^, ^7^ However, for nearly half of the patients with PD‐L1‐negative NSCLC, there is an unmet need for treatment.^8^ A phase III clinical trial (KEYNOTE‐189) demonstrated a possible survival benefit of pembrolizumab combined with chemotherapy in PD‐L1‐negative patients.^7^ Regrettably, the combination of atezolizumab and chemotherapy did not prolong overall survival (OS) in the same population.^9^

In addition, bevacizumab plus first‐line chemotherapy has resulted in remarkable gains in patient outcome for those with advanced NSCLC.^10^ Therefore, in the current study, we evaluated the clinical effects and adverse events (AEs) of patients with PD‐L1 (‐) advanced nonsquamous NSCLC (nNSCLC) who accepted first‐line chemotherapy plus immunotherapy versus antiangiogenic therapy. These results can provide the basis for clinicians' treatment decisions or the conduct of clinical trials.

## 2 METHOD

### 2.1 Study design

**FIGURE 1:**
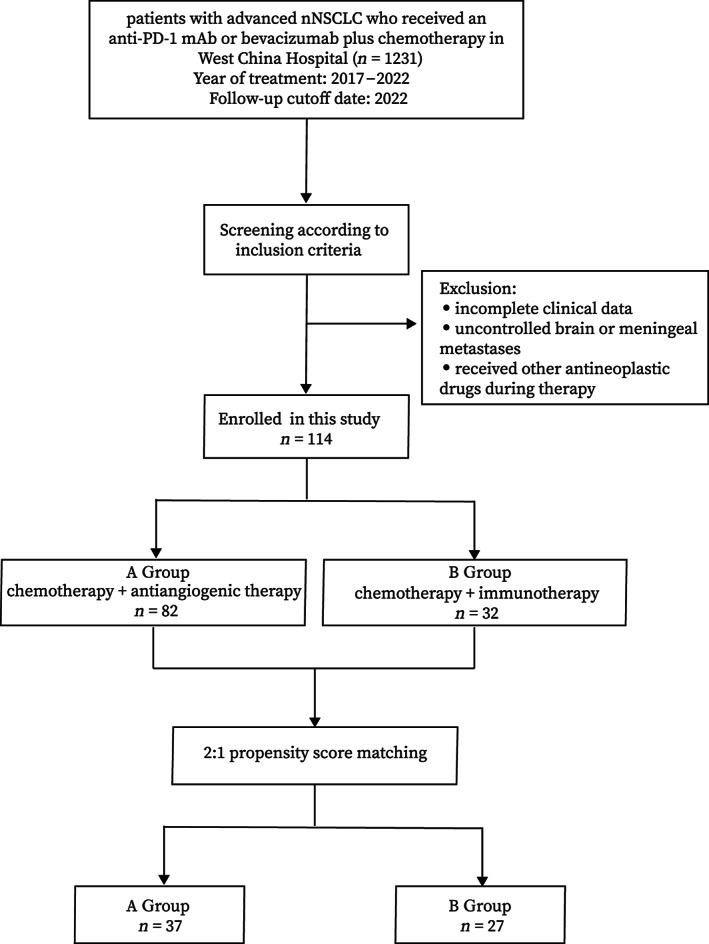
Flow diagram of selecting patients. A Group, patients received pemetrexed, a platinum drug, and bevacizumab; B Group, patients received pemetrexed, a platinum drug, and pembrolizumab/camrelizumab.

The design of the whole study is shown in Figure 1. This study retrospectively collected patients with advanced nNSCLC who received an anti‐PD‐1 mAb or bevacizumab plus chemotherapy from April 3, 2017, to February 12, 2022, at the Department of Thoracic Oncology, West China Hospital. The study was approved by the Institutional Ethics Review Committee of West China Hospital, Sichuan University, Chengdu, China [Approval number: 2022–172]. All patients signed informed consents for chemotherapy, immunotherapy, or antivascular therapy.

Among the inclusion criteria were (1) the age of ≥18 years; (2) pathologically confirmed advanced nNSCLC based on validation of specimens obtained by the guidance of endoscopy or intervention with at least one measurable lesion in accordance with the Response Evaluation Criteria in Solid Tumors (v1.1)^11^; (3) PD‐L1 expression (tumor proportion score) <1%; (4) naïve to first‐line platinum‐containing chemotherapy; (5) could receive immunotherapy or targeted antiangiogenic therapy. Patients were excluded if they had incomplete clinical data, had uncontrolled brain or meningeal metastases, or received other antineoplastic drugs during therapy.

### 2.2 Treatment

Included patients were given 4 or 6 3‐week cycles of pemetrexed (500 mg/m^2^, Sichuan Huiyu Pharmaceutical Co., Ltd.) plus cisplatin (75 mg/m^2^, Qilu Pharmaceutical Co., Ltd.). Pemetrexed (500 mg/m^2^) was continued every 3 weeks sequentially. The whole patient accepted standard preconditioning prior to administration of pemetrexed with 400 μg/d of folic acid orally, 1 mg of vitamin B12 intramuscularly every 3 months, and 3.75 mg/bid of dexamethasone orally. Patients who received bevacizumab (Roche) at 7.5 mg/kg plus pemetrexed and cisplatin in 3‐week cycles were assigned to Group A. Pembrolizumab (Merck Frosst) or camrelizumab (Hengrui) at 200 mg plus pemetrexed and cisplatin were administered on the first day of each 3‐week cycle to patients assigned to arm B. After the intensive induction period, a continuation of administration for pembrolizumab, camrelizumab, or bevacizumab was provided to patients until uncontrolled toxicity or disease progression occurred.

### 2.3 Follow‐up and data collection

Each cycle's follow‐up was performed by the outpatient clinic or by telephone. Patients underwent chest computed tomography (CT), abdominal CT, a cerebral contrast enhanced magnetic resonance scan (MRI) every two cycles, and a bone scan every 6 months. The last follow‐up was conducted on April 24, 2022.

Pretreatment evaluations in both groups include basic information, medical history, physical examination, blood routine examination, serum biochemical tests, bronchoscopy, pathology report, genetic test report, chest or abdominal CT, cerebral MRI, and bone scan from clinical records.

### 2.4 Endpoint's definition

The primary outcome was progression‐free survival (PFS). PFS was measured from the start of treatment until the time of disease progression or death from any cause. The secondary endpoints were determined to include OS, ORR, DCR, and AEs. OS was considered the time between the initiation of treatment and death from any cause. ORR was identified as a complete response (CR) plus partial response (PR), and DCR was characterized as a CR + PR + stable disease (SD). Tumor response was evaluated according to RECIST 1.1 criteria. AEs were appraised on the basis of the National Cancer Institute's Common Terminology Criteria for Adverse Events Version 5.0.18.

### 2.5 Statistical analysis

SPSS 25.0 software (IBM Corp.) and R software version 4.1.1 were both used to update all statistical analyses. Continuous variables were presented as medians (ranges) and assessed through the Mann–Whitney U test. Categorical variables were expressed as percentages and applied for the chi‐squared test. Few PD‐L1‐negative patients are currently treated with immunotherapy, and to minimize the potential confounding effect and to derive well‐matched cohorts, a 1:2 propensity score matching (PSM) approach was used to match different patients who underwent chemotherapy combined with anti‐vascular therapy with patients who underwent chemotherapy combined with immunotherapy. A non‐replacement nearest neighbor matching algorithm was used to ensure a suitable match. Life curves were calculated by the Kaplan–Meier method and measured using the log‐rank test. A *p*‐value <0.05 in a two‐tailed test was considered a statistically notable difference.

## 3 RESULTS

### 3.1 Patient characteristics

The current study contained 114 patients: 82 in Group A and 32 in Group B. A total of 70 men (61.4%) and 44 women (38.6%) were included in the research. The median age of the overall population was 59 years (24–78). 28.9% of the patients were current or former smokers. A slightly higher incidence of common gene mutations was demonstrated in Group A. Baseline demographic and disease characteristics between the two groups were generally well balanced, as shown in Table 1.

**TABLE 1:** Demographic and disease characteristics of the patients at baseline^a^.

Characteristic	A Group (*n* = 82)	B Group (*n* = 32)	*p* ^b^
Age			
Median (range)	57 (24.0–78.0)	61(34.0–77.0)	0.227
Gender, no. (%)			
Male	48 (58.5)	22 (68.8)	0.314
Female	34 (41.5)	10 (32.3)	
ECOG PS, no. (%)^c^			
0	39 (47.6)	12 (37.5)	0.448
1	34 (41.5)	14 (48.3)	
2	9 (11)	6 (18.8)	
Smoking history, no. (%)			
Current or former	22 (26.8)	11 (34.4)	0.425
Never	60 (73.2)	21 (65.6)	
Grade			
Well differentiated	9 (11.0)	4 (12.5)	0.920
Moderately differentiated	9 (11.0)	4 (12.5)	
Poorly differentiated	1 (1.2)	0	
Undifferentiated	0	0	
Unknown	63 (76.8)	24 (75.0)	
Type of mutation, no. (%)			
None	43 (52.4)	25 (78.1)	0.060
EGFR	23 (28.0)	4 (12.5)	
ALK	6 (7.3)	0	
ROS	0	0	
KRAS	10 (12.2)	3(9.4)	
Stage, no. (%)			
IIIB	3 (3.7)	2 (6.3)	0.802
IIIC	2 (2.4)	1 (3.1)	
IV	78 (93.9)	29 (90.6)	
Distant metastasis, no. (%)			
Liver	12 (14.6)	2 (6.3)	0.901
Bone	39 (47.6)	13 (40.6)	
Brain	19 (23.2)	8 (25.0)	
Adrenal	10 (12.2)	4 (12.5)	
Lung	50 (61.0)	15 (46.9)	
Other	25 (30.5)	11 (34.4)	
None	6 (7.3)	3 (9.4)	
Other treatments, no. (%)			
Surgery	28 (34.1)	7 (21.9)	0.305
Radiotherapy	34 (41.5)	10 (31.3)	
Targeted therapy	29 (35.4)	3 (9.4)	

^a^ Patients in arm A received pemetrexed, a platinum drug, and bevacizumab; patients in arm B received pemetrexed, a platinum drug, and pembrolizumab/camrelizumab. The percentages may not add up to 100 due to rounding.

^b^ At a two‐sided alpha level of 0.05, there were no significant differences between the groups on all baseline characteristics.

^c^ Eastern Cooperative Oncology Group (ECOG) and Performance Status Score (PS) performance status scores range from 0 to 5, with 0 being asymptomatic and higher scores indicating greater disability.

Abbreviations: ECOG, Eastern Cooperative Oncology Group; PD‐L1, programmed death ligand 1; PS, performance status; TPS, tumor proportion score.

### 3.2 Survival analyses before PSM

**FIGURE 2:**
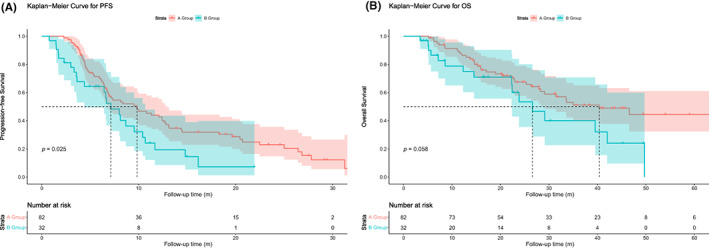
Kaplan–Meier curves for PFS and OS in total Groups A and B populations. (A) Kaplan–Meier curves for PFS in total Groups A and B populations; (B) Kaplan–Meier curves for OS in total Groups A and B populations. A Group, patients received pemetrexed, a platinum drug, and bevacizumab; B Group, patients received pemetrexed, a platinum drug, and pembrolizumab/camrelizumab; OS, overall survival; PFS, progression‐free survival.

The median follow‐up time in this study was 35.0 months (3.4–136.1 months). For the total population, the median PFS (mPFS) was 8.1 months (95% confidence interval [CI]: 5.9–10.3). At last follow‐up, disease progression occurred in 83 patients (76.3%), of whom 63 (76.8%) were in Group A and 24 (75%) in Group B. The mPFS of the patients in Group A was 9.8 months (95% CI: 6.0–13.7) and in Group B was 6.7 months (95% CI: 5.5–7.8), respectively (Figure 2A), and there is a statistical difference between the groups (*p* = 0.025). The median OS (mOS) was 40.4 months (95% CI: 25.0–55.8) and 26.6 months (95% CI: 19.0–34.2) for patients in Groups A and B (*p* = 0.058), respectively (Figure 2B).

### 3.3 Survival analyses after PSM

**FIGURE 3:**
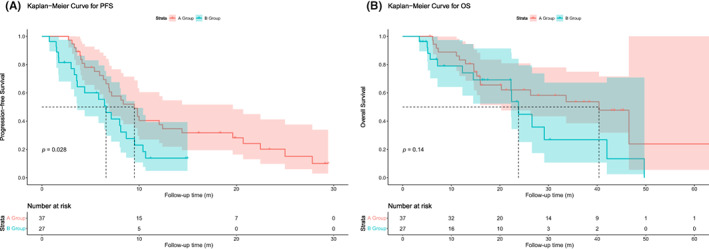
Kaplan–Meier curves of PFS and OS for patients in Group A and Group B after propensity score matching .(A) Kaplan–Meier curves for PFS in Group A and Group B after propensity score matching; (B) Kaplan–Meier curves for OS in Group A and Group B after propensity score matching. OS, overall survival; PFS, progression‐free survival.

Further analyses were performed in two independent post‐match cohorts (Group A and Group B matched 2:1) using PSM analysis for sex, age, surgery, ECOG, differentiation, metastasis, and radiotherapy. A strictly matched group of 37 individuals in Group A and 27 individuals in Group B was used for the follow‐up survival analysis. The mPFS was 12.9 months (95% CI: 9.9–16.0) for patients in Group A and 6.9 months (95% CI: 5.1–8.6) in Group B after PSM, with a significant difference in mPFS between the two groups (*p* = 0.028) (Figure 3A). The mOS was 36.7 months (95% CI: 27.0–46.4) and 25.7 months (95% CI: 18.0–32.7) for patients in Groups A and B (*p* = 0.14), respectively (Figure 3B).

### 3.4 Tumor response

The therapeutic effect of the two regimens is displayed in Table 2. Among 114 patients, therapy was initiated and accomplished in at least two therapeutic cycles. Of the 82 patients administered bevacizumab combined with medical cure, one of these cases achieved CR, 38 cases reached PR, and the best efficacy of the 38 cases was SD. ORR and DCR for Group A were 52.4% and 93.9%, respectively. Ten patients discontinued treatment due to COVID‐19 and economic reasons. In the combined immunotherapy group, no patients achieved CR, but the treatment achieved an ORR and a DCR comparable to those of the A Group, with an ORR of 50.0% and a DCR of 87.5%. Due to COVID‐19, two patients discontinued treatment. No statistically meaningful differences were obtained in ORR or DCR between Groups A and B.

**TABLE 2:** Summary of treatment efficacy in patients who underwent tumor evaluation.

Treatment efficacy, *n* (%)	A Group (*n* = 82)	B Group (*n* = 32)	*p*
Complete response	1 (1.2)	0	
Partial response	38 (46.3)	16 (50.0)	
Stable disease	38 (46.3)	12 (37.5)	
Progressive disease	0	3(9.4)	
Could not be evaluated	5 (6.1)	1 (3.1)	
Objective response rate	39 (52.4)	16 (50.0)	0.815
Disease control rate	77 (93.9)	28 (87.5)	0.225

Abbreviations: A group, antiangiogenic agents + chemotherapy; B group, Immune drugs + chemotherapy.

### 3.5 Subgroup analysis

**FIGURE 4:**
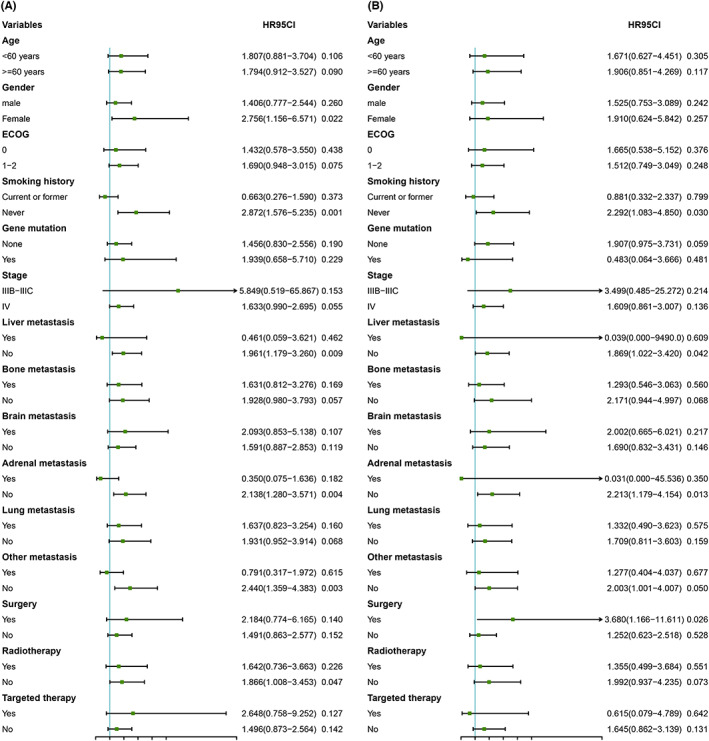
Subgroup analyses for PFS (A) and OS (B). CI, confidence interval; ECOG, Eastern Cooperative Oncology Group; HR, hazard ratio.

Moreover, to better define the prognosis between Groups A and B, we conducted a subgroup analysis of PFS and OS within the cohort (Figure 4). In terms of patient PFS, patients in the A Group achieved a better clinical outcome with the following factors: female sex, never smoking, absence of liver metastasis, absence of adrenal metastasis, and absence of uncommon metastasis. For OS, patients in the A Group who did not smoke, did not have adrenal metastases, or underwent surgery could benefit from survival. For other parameters not mentioned above, no statistical difference was obtained between the two groups in PFS and OS.

### 3.6 Adverse events

Almost all AEs are summarized in Table 3. The majority of patients underwent AEs resulting from cancer‐related treatments. In general, AEs in Group A were more common than those in Group B. No patients in either group experienced Grade 5 AEs. Treatment‐related Grade III–V AEs appeared in 16 patients (19.5%) in the A Group and 5 patients (15.6%) in the B Group. AEs leading to treatment discontinuation occurred in four patients (4.9%) in the A Group and 3 (9.4%) in the B Group. In the bevacizumab‐combined group, the most frequently occurring AEs were anemia (43.9%), leukopenia (40.2%), neutropenia (25.6%), and fatigue (24.4%). The most significant grade III–V AEs were neutropenia (4.8%) and thrombus (3.7%). Likewise, in the immunotherapy‐combined group, the most common AEs were anemia (59.3%) and leukopenia (46.9%). Hepatitis (6.3%) was the most frequent grade III‐V AE. Notably, four patients had hand‐foot syndrome in Group A. Thyroid dysfunction and pneumonitis were found in the Group B population.

**TABLE 3:** Adverse events of groups.

Adverse events, *n* (%)	A Group (*n* = 82)		B Group (*n* = 32)		*p*
Treatment‐related AEs					
Any grade	61 (74.4)		25 (78.1)		
Grade III‐V	16 (19.5)		5 (15.6)		
Serious AEs	2 (2.4)		0		
Led to discontinuation	4 (4.9)		3 (9.4)		
Led to death	0		0		
Treatment‐related AEs in either arm	I‐II	III‐IV	I‐II	III‐IV	
Leukopenia	33 (40.2)	2 (2.4)	15 (46.9)	0	0.579
Neutropenia	21 (25.6)	4 (4.9)	8 (25.0)	1 (3.1)	0.913
Anemia	36 (43.9)	2 (2.4)	19 (59.3)	1 (3.1)	0.300
Thrombocytopenia	14(17.1)	2 (2.4)	3 (9.4)	0	0.258
Fatigue	20 (24.4)	0	10 (31.0)	0	0.455
Nausea/vomiting	18 (22.0)	0	6 (18.8)	0	0.706
Diarrhea	0	0	1 (3.1)	0	0.108
Hepatitis	13 (15.9)	1 (1.2)	5 (15.6)	2 (6.3)	0.320
Renal insufficiency	3 (3.7)	0	4 (12.5)	0	0.077
Thrombus	4 (4.9)	3 (3.7)	0	0	0.233
Rash	1 (1.2)	0	1 (3.1)	0	0.486
Bleeding	1 (1.2)	0	0	0	0.530
Hand‐foot syndrome	4 (4.9)	0	0	0	0.203
Hypothyroidism	2 (2.4)	0	2 (6.3)	1 (3.1)	0.163
Pneumonitis	0	0	3 (9.4)	0	0.005
Hyperthyroidism	0	0	2 (6.3)	0	0.022

Abbreviations: AE, adverse event; A group, antiangiogenic agents + chemotherapy; B group, immune drugs + chemotherapy.

## 4 DISCUSSION

For certain patients with advanced or locally advanced lung cancer who are eligible for immunotherapies or targeted therapies, the 5‐year survival rate has been effectively prolonged compared to patients with traditional platinum‐containing 2‐drug chemotherapy.^12^, ^13^, ^14^ Pembrolizumab, camrelizumab, and bevacizumab all have antitumor effects, and their benefit in the therapy of advanced nNSCLC has been demonstrated.^7^, ^15^, ^16^ Nevertheless, the survival benefit of immunotherapy combined with chemotherapy in patients with PD‐L1(‐) nNSCLC is unclear.^7^, ^17^ Therefore, we retrospectively explored the efficacy and AEs of patients with PD‐L1(‐) advanced nNSCLC who received immunotherapy combined with chemotherapy versus a combination of antiangiogenic therapy and chemotherapy. In the baseline table of patients, the number of EGFR mutations in Group A was twice that of Group B. And, bevacizumab improved PFS in patients with common EGFR mutations.^18^ PFS was improved in patients with rare EGFR mutations treated with immunotherapy compared with those with sensitive EGFR mutations.^19^ This can affect group‐to‐group comparability, which is why we use PSM. The results demonstrated that in advanced nNSCLC patients with levels of PD‐L1 < 1%, bevacizumab plus chemotherapy has a PFS advantage over immunotherapy plus chemotherapy with controllable AEs, both before and after PSM. In terms of OS, ORR, and DCR, the two treatment regimens are comparable. It is worth noting that the differences in OS between Group A and Group B were almost statistically significant (*p* = 0.06) before the PSM was performed, and after the PSM, there was still no statistical difference between the OS between Group A and Group B (*p* = 0.14).

Previous large clinical trials confirm that bevacizumab plus carboplatin/paclitaxel can improve clinical outcomes for patients with advanced nNSCLC.^20^, ^21^, ^22^ A phase II clinical study of bevacizumab plus cisplatin and pemetrexed in patients with wild‐type EGFR advanced non‐squamous NSCLC showed a mPFS of 12.0 months, a mOS of 31.0 months, and an ORR of 70%. Similar to the previous outcomes, our patients in the bevacizumab combination group gained a mPFS of 9.8 months and a mOS of 40.4 months, with an ORR of 52.4%.

Immunomodulators that target PD‐1 or PD‐L1 as new first‐line monotherapy or combination regimens are now recommended in the NCCN Clinical Practice Guidelines in Oncology.^7^; ^9^; ^23^ The phase III clinical trial, CameL, achieved a mPFS of 11.3 months, and the estimated mOS in the group receiving camrelizumab plus chemotherapy was 27.9 months. However, in patients with PD‐L1 levels of <1%, combination therapy with camrelizumab did not bring significant benefit (HR 0.76 [95% CI 0.45–1.26]). Another phase III study, KEYNOTE‐189, identified that the administration of pembrolizumab, pemetrexed, and platinum showed significant prolongation of OS and PFS at diverse PD‐L1 expression levels in patients receiving chemotherapy alone.^7^ In the subgroup analysis of patients at levels of PD‐L1 < 1%, the mPFS and mOS were 9.0 (95% CI: 8.1–9.9) months and 17.2 (95% CI: 13.8–22.8) months,^24^ respectively, similar to our results of a median (95% CI) PFS of 6.7 (5.5–7.8) months and a median (95% CI) OS of 26.6 (19.0–34.2) months. We can see that there is a significant advantage to combined immunotherapy in OS.

In the subgroup analysis, female and never‐smoking patients could achieve better PFS and OS with anti‐vascular combination chemotherapy. This is consistent with the results of a subgroup analysis on bevacizumab after PSM,^25^ for which there was no significant benefit of nivolumab or pembrolizumab compared with chemotherapy at the time of treatment for female patients.^26^, ^27^ In addition, patients who never smoke or smoke lightly may benefit more from anti‐vascular combined chemotherapy.^28^ The benefits of immunotherapy for smokers are significantly greater than for non‐smokers.^29^ The benefits of immunotherapy for smokers are significantly greater than for non‐smokers. A meta‐analysis found that anti‐PD‐1 and chemotherapy tended to benefit PFS for liver transfers.^30^ Our subgroup analysis findings are not contradictory: in patients with no liver transfers, the A‐Group treatment model could benefit from PFS.

In this study, immunotherapy combined with chemotherapy did not show an advantage in PFS but prolonged OS in patients with advanced lung adenocarcinoma at PD‐L1 levels of <1%, with an efficacy comparable to bevacizumab combined with chemotherapy. This may demonstrate the long‐term impact of immunotherapy on patient survival outcomes. In addition, some patients treated with bevacizumab plus chemotherapy as first‐line treatment will receive immunotherapy after disease progression, highlighting the long‐term survival advantage of immunotherapy. Furthermore, patients with a sensitive gene mutation who received targeted therapy after progressing on anti‐vascular therapy combined with chemotherapy may benefit from OS.

The study has the following limitations: First, the sample size was relatively small, and enrollment needs to be expanded to validate our results. Secondly, excluding the limitation of the number of patients, we are better off using pembrolizumab uniformly. Third, all our enrolled patients were Chinese. However, no racial differences have been reported in previously relevant phase III trials, so our results should apply to most ethnic groups. Lastly, this was a nonrandomized retrospective study from a single center with limited generalizability of the results.

Both regimens are recommended as first‐line treatment options for patients with advanced nNSCLC. Clinically, in the light of medical insurance conditions, patients with active autoimmune disease, no bleeding risk, or economic conditions, we can choose bevacizumab plus pemetrexed and platinum as first‐line treatment. In the current study, similar long‐term survival results were obtained, but a slightly higher PFS benefit was obtained in the bevacizumab combination group than in the immunotherapy combination group. Further prospective clinical trials or retrospective studies with large cohorts are expected to prove the findings of this study.

## 5 CONCLUSION

Anti‐PD‐1 antibodies or bevacizumab combined with chemotherapy, followed by related maintenance therapy, had similar OS, ORR, DCR, and AEs in patients with advanced nNSCLC at PD‐L1 levels of <1%. Better PFS was achieved with bevacizumab, pemetrexed, and cisplatin. The administration of immunotherapy or bevacizumab in combination with pemetrexed plus cisplatin was beneficial and well tolerated in PD‐L1‐negative patients with advanced nNSCLC. Bevacizumab combined with pemetrexed and cisplatin is more recommended for first‐line treatment of patients with advanced lung adenocarcinoma at PD‐L1 levels of <1%.

## AUTHOR CONTRIBUTIONS

**Ruolan Xia:** Formal analysis (equal); writing – original draft (equal). **Yanying Li:** Investigation (equal). **Ling Yang:** Writing – original draft (equal). **Meijuan Huang:** Resources (equal).

## FUNDING INFORMATION

None.

## CONFLICT OF INTEREST STATEMENT

The authors declare that they have no known competing financial interests or personal relationships that could have appeared to influence the work reported in this paper.

## Data Availability

Data sharing is not applicable to this article as no new data were created or analyzed in this study.

## References

[1] Thai AA , Solomon BJ , Sequist LV , Gainor JF , Heist RS . Lung cancer. Lancet (London, England). 2021;398:535‐554. 34273294 10.1016/S0140-6736(21)00312-3

[2] <biesalski1998.pdf>.

[3] Ettinger DS , Wood DE , Aisner DL , et al. Non‐small cell lung cancer, version 2.2021. J Natl Compr Canc Netw. 2021;19(3):254‐266. 33668021 10.6004/jnccn.2021.0013

[4] Biesalski HK , Bueno de Mesquita B , Chesson A , et al. European consensus statement on lung cancer: risk factors and prevention. Lung cancer panel. CA Cancer J Clin. 1998;48:167‐176. discussion 164‐6. 9594919 10.3322/canjclin.48.3.167

[5] Reck M , Remon J , Hellmann MD . First‐line immunotherapy for non‐small‐cell lung cancer. J Clin Oncol. 2022;40:586‐597. 34985920 10.1200/JCO.21.01497

[6] Reck M , Rodríguez‐Abreu D , Robinson AG , et al. Pembrolizumab versus chemotherapy for PD‐L1‐positive non‐small‐cell lung cancer. N Engl J Med. 2016;375:1823‐1833. 27718847 10.1056/NEJMoa1606774

[7] Gandhi L , Rodríguez‐Abreu D , Gadgeel S , et al. Pembrolizumab plus chemotherapy in metastatic non‐small‐cell lung cancer. N Engl J Med. 2018;378:2078‐2092. 29658856 10.1056/NEJMoa1801005

[8] Dietel M , Savelov N , Salanova R , et al. Real‐world prevalence of programmed death ligand 1 expression in locally advanced or metastatic non–small‐cell lung cancer: the global, multicenter EXPRESS study. Lung Cancer. 2019;134:174‐179. 31319978 10.1016/j.lungcan.2019.06.012

[9] Socinski MA , Jotte RM , Cappuzzo F , et al. Atezolizumab for first‐line treatment of metastatic nonsquamous NSCLC. N Engl J Med. 2018;378:2288‐2301. 29863955 10.1056/NEJMoa1716948

[10] Zinner RG , Obasaju CK , Spigel DR , et al. PRONOUNCE: randomized, open‐label, phase III study of first‐line pemetrexed + carboplatin followed by maintenance pemetrexed versus paclitaxel + carboplatin + bevacizumab followed by maintenance bevacizumab in patients ith advanced nonsquamous non‐small‐cell lung cancer. J Thorac Oncol. 2015;10:134‐142. 25371077 10.1097/JTO.0000000000000366 PMC4276572

[11] Eisenhauer EA , Therasse P , Bogaerts J , et al. New response evaluation criteria in solid tumours: revised RECIST guideline (version 1.1). Eur J Cancer. 2009;45:228‐247. 19097774 10.1016/j.ejca.2008.10.026

[12] Garon EB , Hellmann MD , Rizvi NA , et al. Five‐year overall survival for patients with advanced non–small‐cell lung cancer treated with pembrolizumab: results from the phase I KEYNOTE‐001 study. J Clin Oncol. 2019;37:2518‐2527. 31154919 10.1200/JCO.19.00934 PMC6768611

[13] Lin JJ , Cardarella S , Lydon CA , et al. Five‐year survival in EGFR‐mutant metastatic lung adenocarcinoma treated with EGFR‐TKIs. J Thoracic Oncol. 2016;11:556‐565. 10.1016/j.jtho.2015.12.103 PMC4979601 26724471

[14] Zhao D , Chen X , Qin N , et al. The prognostic role of EGFR‐TKIs for patients with advanced non‐small cell lung cancer. Sci Rep. 2017;7:40374. 28079142 10.1038/srep40374 PMC5227705

[15] Garcia J , Hurwitz HI , Sandler AB , et al. Bevacizumab (Avastin®) in cancer treatment: a review of 15 years of clinical experience and future outlook. Cancer Treat Rev. 2020;86:102017. 32335505 10.1016/j.ctrv.2020.102017

[16] Zhou C , Chen G , Huang Y , et al. Camrelizumab plus carboplatin and pemetrexed versus chemotherapy alone in chemotherapy‐naive patients with advanced non‐squamous non‐small‐cell lung cancer (CameL): a randomised, open‐label, multicentre, phase 3 trial. Lancet Respir Med. 2021;9:305‐314. 33347829 10.1016/S2213-2600(20)30365-9

[17] Luo H , Lu J , Bai Y , et al. Effect of Camrelizumab vs placebo added to chemotherapy on survival and progression‐free survival in patients with advanced or metastatic esophageal squamous cell carcinoma: the ESCORT‐1st randomized clinical trial. JAMA. 2021;326:916‐925. 34519801 10.1001/jama.2021.12836 PMC8441593

[18] Yang JC . Bevacizumab in EGFR‐positive NSCLC: time to change first‐line treatment? Lancet Oncol. 2019;20:602‐603. 30975629 10.1016/S1470-2045(19)30085-3

[19] Shi C , Wang Y , Xue J , Zhou X . Immunotherapy for EGFR‐mutant advanced non‐small‐cell lung cancer: current status, possible mechanisms and application prospects. Front Immunol. 2022;13:940288. 35935943 10.3389/fimmu.2022.940288 PMC9353115

[20] Sandler A , Gray R , Perry MC , et al. Paclitaxel‐carboplatin alone or with bevacizumab for non‐small‐cell lung cancer. N Engl J Med. 2006;355:2542‐2550. 17167137 10.1056/NEJMoa061884

[21] Reck M , von Pawel J , Zatloukal P , et al. Phase III trial of cisplatin plus gemcitabine with either placebo or bevacizumab as first‐line therapy for nonsquamous non‐small‐cell lung cancer: AVAil. J Clin Oncol. 2009;27:1227‐1234. 19188680 10.1200/JCO.2007.14.5466

[22] Zhou C , Wu YL , Chen G , et al. BEYOND: a randomized, double‐blind, placebo‐controlled, multicenter, phase III study of first‐line carboplatin/paclitaxel plus bevacizumab or placebo in Chinese patients with advanced or recurrent nonsquamous non‐small‐cell lung cancer. J Clin Oncol. 2015;33:2197‐2204. 26014294 10.1200/JCO.2014.59.4424

[23] Mok TSK , Wu YL , Kudaba I , et al. Pembrolizumab versus chemotherapy for previously untreated, PD‐L1‐expressing, locally advanced or metastatic non‐small‐cell lung cancer (KEYNOTE‐042): a randomised, open‐label, controlled, phase 3 trial. Lancet. 2019;393:1819‐1830. 30955977 10.1016/S0140-6736(18)32409-7

[24] Gadgeel S , Rodríguez‐Abreu D , Speranza G , et al. Updated analysis from KEYNOTE‐189: pembrolizumab or placebo plus pemetrexed and platinum for previously untreated metastatic nonsquamous non‐small‐cell lung cancer. J Clin Oncol. 2020;38:1505‐1517. 32150489 10.1200/JCO.19.03136

[25] Qi F , Hu X , Liu Y , et al. First‐line pemetrexed‐platinum doublet chemotherapy with or without bevacizumab in non‐squamous non‐small cell lung cancer: a real‐world propensity score‐matched study in China. Chin J Cancer Res. 2019;31:749‐758. 31814679 10.21147/j.issn.1000-9604.2019.05.05 PMC6856705

[26] Pinto JA , Vallejos CS , Raez LE , et al. Gender and outcomes in non‐small cell lung cancer: an old prognostic variable comes back for targeted therapy and immunotherapy? ESMO Open. 2018;3:e000344. 29682332 10.1136/esmoopen-2018-000344 PMC5905840

[27] Vavalà T , Catino A , Pizzutilo P , Longo V , Galetta D . Gender differences and immunotherapy outcome in advanced lung cancer. Int J Mol Sci. 2021;22(21): 11942. 34769372 10.3390/ijms222111942 PMC8584562

[28] Weiss JM , Villaruz LC , O'Brien J , et al. Results of a phase II trial of carboplatin, pemetrexed, and bevacizumab for the treatment of never or former/light smoking patients with stage IV non‐small cell lung cancer. Clin Lung Cancer. 2016;17:128‐132. 26774201 10.1016/j.cllc.2015.12.006 PMC4844001

[29] Li X , Huang C , Xie X , et al. The impact of smoking status on the progression‐free survival of non‐small cell lung cancer patients receiving molecularly target therapy or immunotherapy versus chemotherapy: a meta‐analysis. J Clin Pharm Ther. 2021;46:256‐266. 33152129 10.1111/jcpt.13309

[30] Chen J , Liu X , Zhang J , et al. Frontline anti‐PD‐1/PD‐L1 versus bevacizumab in advanced non‐small‐cell lung cancer: a network meta‐analysis. Future Oncol. 2022;18:1651‐1664. 35129371 10.2217/fon-2021-0838

